# The Effect of Supplementation with Low Doses of a Cod Protein Hydrolysate on Satiety Hormones and Inflammatory Biomarkers in Adults with Metabolic Syndrome: A Randomized, Double-Blind Study

**DOI:** 10.3390/nu12113421

**Published:** 2020-11-08

**Authors:** Caroline Jensen, Hanna Fjeldheim Dale, Trygve Hausken, Jan Gunnar Hatlebakk, Ingeborg Brønstad, Gülen Arslan Lied, Dag Arne Lihaug Hoff

**Affiliations:** 1 Centre for Nutrition, Department of Clinical Medicine, University of Bergen, 5021 Bergen, Norway; hanna.dale@outlook.com (H.F.D.); trygve.hausken@helse-bergen.no (T.H.); jan.gunnar.hatlebakk@helse-bergen.no (J.G.H.); gulen.arslan@uib.no (G.A.L.); 2 Division of Gastroenterology, Department of Medicine, Haukeland University Hospital, 5021 Bergen, Norway; ingeborg.bronstad@helse-bergen.no; 3 National Centre of Functional Gastrointestinal Disorders, Haukeland University Hospital, 5021 Bergen, Norway; 4 Division of Gastroenterology, Department of Medicine, Ålesund Hospital, Møre and Romsdal Hospital Trust, 6026 Ålesund, Norway; dag.arne.lihaug.hoff@helse-mr.no; 5 Department of Clinical and Molecular Medicine, Faculty of Medicine and Health Science, Norwegian University of Science and Technology, 7491 Trondheim, Norway

**Keywords:** cod protein hydrolysate, satiety hormones, inflammatory markers, metabolic syndrome

## Abstract

Metabolic syndrome (MetS) is characterised by metabolic abnormalities that increase the risk of developing type 2 diabetes mellitus and cardiovascular disease. Altered levels of circulating ghrelin, several adipokines and inflammatory markers secreted from adipose tissue, such as leptin, adiponectin, tumor necrosis factor alpha, are observed in overweight and obese individuals. We assessed the effect of supplementation with low doses of a cod protein hydrolysate (CPH) on fasting and postprandial levels of acylated ghrelin, as well as fasting levels of adiponectin, leptin and inflammatory markers in subjects with MetS. A multicentre, double-blinded, randomized controlled trial with a parallel group design was conducted. Subjects received a daily supplement of CPH (4 g protein, *n* = 15) or placebo (0 g protein, *n* = 15). We observed no effect on fasting or postprandial levels of acylated ghrelin, fasting levels of adiponectin (*p* = 0.089) or leptin (*p* = 0.967) after supplementation with CPH, compared to placebo. Overall, our study showed that 8 weeks supplementation with a low dose of CPH in subjects with MetS had no effect on satiety hormones or most of the inflammatory markers, but the levels of high-sensitivity C-reactive protein were statistically significantly different in the CPH-group compared to placebo group. The robustness and clinical relevance of these findings should be explored in future studies with a larger sample size.

## 1. Introduction

Metabolic syndrome (MetS) represents a cluster of metabolic abnormalities including abdominal obesity, hypertriglyceridemia, low levels of high-density cholesterol, hyperglycemia and hypertension, increasing the risk of cardiovascular disease and type 2 diabetes mellitus (T2DM) [1,2]. The pathogenesis of MetS is not fully understood, but a genetic predisposition combined with a sedentary lifestyle and excessive caloric intake, are known to be important risk factors [3]. A positive energy balance over time leads to increased storage of fat in the adipocytes with expansion of adipose tissue, resulting in increased production of pro-inflammatory cytokines such as interleukin 6 (IL-6) and tumor necrosis factor alpha (TNF-α) [3,4]. These cytokines and other signaling molecules secreted from the adipose tissue are involved in several physiological processes in the body, such as regulation of energy homeostasis, body fat accumulation and inflammation [5,6,7].

Inflammation is the immune system’s response to an injurious stimulus, initiating a cascade of events promoting healing of the affected tissue [8]. Signaling pathways are activated as part of the inflammatory response, leading to the release of inflammatory mediators, including interleukin-1 beta (IL-1β), IL-6 and TNF-α [9,10]. Even though the acute inflammatory response is essential, the process may develop to a chronic inflammatory state, known to be involved in the development of several chronic diseases, such as diabetes [9]. Fish consumption has been linked to reduced levels of inflammatory mediators, such as IL-6 and TNF-α in rats [11,12] and healthy adults [13]. Lower levels of C-reactive protein (CRP), a marker of inflammation, have been observed in insulin-resistant subjects given a diet with lean fish (cod) for 4 weeks [14], with no effect observed when overweight individuals were given a low dose of cod protein supplementation [15]. The mechanism behind the possible anti-inflammatory effect of fish is largely unknown, but it has been suggested that the high taurine content in fish may have anti-inflammatory properties by suppressing IL-6 and TNF-α [12,16]. There are also indications from cell and animal studies, that bioactive peptides from lean fish have anti-inflammatory effects [9,17], with a need for further investigation in humans.

The secretion of IL-6, IL-8 and other cytokines are inhibited by adiponectin, a signaling molecule released from adipose tissue [4,18]. This molecule is known to enhance insulin sensitivity [4], and reduced levels have been reported in subjects with obesity and T2DM [5,19]. In contrast, increased levels of leptin, a hormone involved in regulation of food intake and appetite [7,20], have been reported in obese subjects [19]. Furthermore, consumption of fish and supplementation with *n*-3 polyunsaturated fatty acids (PUFAs) increase levels of adiponectin [19,21,22], with similar results reported after supplementation with cod protein in overweight and obese subjects [23]. For leptin, published data are conflicting [19,21,23,24]. As leptin and adiponectin seem to be altered in subjects with obesity and associated with factors of MetS, an effect on these adipokines by supplementation with fish protein, might be a possible preventive strategy for the development of MetS.

Ghrelin, a small peptide hormone secreted from the stomach, is an appetite-stimulating hormone with the opposite effects of leptin [25]. The levels of ghrelin increase before a meal and decrease postprandially [26], and is involved in regulation of appetite, energy balance and body weight [27]. Compounds that may inhibit the action of ghrelin and suppress appetite, may be beneficial for both prevention and treatment of components of MetS, such as obesity, impairments in lipid metabolism or glucose homeostasis [28]. We have previously reported the effect of low doses of a cod protein hydrolysate (CPH) on fasting and postprandial levels of acylated ghrelin in healthy adults [29]. We observed that a single dose of 20 mg/kg body weight of CPH did not affect postprandial levels of acylated ghrelin or sensations related to feeling of hunger, when compared to the control group [29].

Limited data exists on long-term supplementation of cod protein in a population with metabolic abnormalities, yet it is an important abnormality to study. We have previously reported on the effects on fasting and postprandial glucose metabolism, as well as lipid metabolism and body composition in subjects with MetS after supplementation with 4 g of CPH for 8 weeks [30]. In the present study, we aimed to investigate if daily supplementation with the same low dose of CPH for 8 weeks would influence circulating levels of ghrelin, adiponectin, leptin, high-sensitivity CRP (hs-CRP) and a selection of other inflammatory markers.

## 2. Materials and Methods

### 2.1. Study Design

We performed a multicenter, double-blinded, randomized parallel group trial with one daily dose of 4 g CPH or placebo for 8 weeks. Here, we report secondary outcomes; fasting and postprandial levels of acylated ghrelin and fasting levels of adiponectin, leptin and inflammatory markers. The study was conducted according to the Declaration of Helsinki and all procedures were approved by the Regional Committee for Medical and Health Research Ethics of Central Norway (2018/2163). Written informed consent was obtained from all subjects. The trial is registered at www.clinicaltrials.gov (NCT03807752).

### 2.2. Subjects and Study Setting

We recruited participants between March and September 2019 in the Bergen and Ålesund area (Norway) through an online recruitment questionnaire with advertisements on social media, at the participating hospitals and at general practitioners’ offices. Inclusion criteria were diagnosis of MetS, body mass index (BMI) between 27–35 kg/m^2^ and age between 40–70 years. Exclusion criteria were intolerance or allergy to fish and/or shellfish, chronic diseases or medication that were likely to interfere with the evaluation of study endpoints (e.g., T2DM, medications known to affect glucose metabolism), acute infections, abuse of alcohol or drugs (assessed by a physician) or unwillingness to comply with the study requirements. We included participants using calcium channel blockers (*n* = 3) agents acting on the renin-angiotensin system (i.e., ACE inhibitor *n* = 2, AII-receptor agonist *n* = 5, AII-receptor agonist/thiazide diuretic *n* = 3), since blood pressure was not an outcome. The participants were excluded if they had recently started with the current medication, if they had changed the dose level during the last 3 months or if it was changed during the study. Subjects using beta-blocking agents or peripheral vasodilators were excluded.

In this study, the Joint Interim Statement definition of MetS was used [1], where three abnormal findings out of five given risk factors qualifies for a diagnosis of MetS. The following risk factors and criteria were used: serum triglycerides ≥ 1.7 mmol/L, high-density lipoprotein cholesterol < 1.0 mmol/L in men and < 1.3 mmol/L in women, serum glucose ≥ 5.5 mmol/L, systolic blood pressure ≥ 130 mmHg and/or diastolic blood pressure ≥ 85 mmHg [1]. For waist circumference (WC), we used the International Diabetes Federation cut-off points for central obesity: WC ≥ 94 cm in men, ≥ 80 cm in women [31].

### 2.3. Study Visits

After prescreening by telephone, we invited potential participants to a screening visit to assess eligibility, including inclusion and exclusion criteria. The screening visit included a clinical examination by a physician, review of medical history, measure of vital signs (blood pressure, heart rate), anthropometric measures (height, weight and WC) and blood sampling. We measured height (to the nearest 0.1 cm) and weight (to the nearest 0.1 kg) with an electronic scale (Seca 285, SECA GmbH, Hamburg, Germany). For measurements of WC, the WHO recommendation was followed [32,33]; the midpoint between the lower/inferior palpable rib and the top of iliac crest was located, the participants had arms relaxed at the side and the measurements were made at the end of normal expiration, using a measuring tape with constant tension. Changes in physical activity during the study period, as well as changes to food consumption were prohibited. The participants recorded their intake of food and drink in a three-day prospective food diary, before the baseline visit and end of study visit. Energy and protein intake from the supplement were added to the end of study dietary records (CPH group: 44 kcal, 4 g protein; placebo group: 46.5 kcal, 0 g protein). Details about the end of study energy intake is reported in a previous publication [30]. Calculations of energy and macronutrient intake were determined using “*Kostholdsplanleggeren*” (Norwegian Food Safety Authority, Norwegian Directorate of Health, Oslo) [34]. The participants had to stop the use of *n-3* PUFA-containing dietary supplements for four weeks prior to starting the study, and this was prohibited for the duration of the study.

After inclusion, the participants attended two identical study visits: the baseline visit and end of study visit (after 8 weeks of intervention). The participants came to the research facility between 08:00 AM–09:00 AM in a fasting state (no eating, drinking or use of any nicotine-containing substance after 09:00 PM the previous evening). When they arrived, fasting blood samples were taken followed by anthropometric measurements. Body composition was measured by a bioelectrical impedance analysis device (Body Composition Analyzer, Tanita Corporation, Tokyo, Japan, BC-418 MA (model used in Ålesund), or MC-180 MA (model used in Bergen)) according to the manufacturer’s instructions. The participants then consumed a standardized breakfast meal (test meal) consisting of two slices of semi-dark bread (50% whole wheat, 80 g weight), 10 g margarine, 25 g strawberry jam and 20 g white cheese and 1.5 dL orange juice. The meal contained 1840 kJ (440 kcal), 69 g carbohydrate, 13.3 g protein, 14.3 g fat. The energy and macronutrient content of the test meal were calculated using *“Kostholdsplanleggeren”* [34]. The meal had to be consumed within 15 min and was followed by postprandial blood sampling. Blood was drawn from an antecubital vein prior to the test meal (−20 min), at 0 min (i.e., immediately after the meal was consumed), and thereafter at 20, 40, 60, 80, 100 and 120 min. No coffee or tea were served during test hours, but we allowed free drinking of water. We handed 8 weeks supply of the pre-packed test material (active or placebo) to the participants at the end of the baseline study visit. They started the intervention on the following day and took the supplement daily, 10 min before breakfast, for 8 weeks. Since the participants met fasting at both study visit, they did not take the study supplement at home before the end of study visit.

### 2.4. Test Material

The test material was manufactured by Firmenich Bjørge Biomarin AS (Ålesund, Norway), and delivered pre-packed in sealed plastic-coated aluminium bags. It was a lemon-flavored powder to be mixed with 100 mL cold water before ingestion. The powder bags with intervention material (CPH) contained 4 g of hydrolysed cod protein, 5 g glucose hydrate, 2 g maltodextrin, 0.025 g tastegram powder flavour, 0.1 g lemongrass durarome taste and 0.7 g citric acid. The placebo contained 6.5 g of maltodextrin, 0.2 g citric acid, and was otherwise identical to the intervention material. It was not possible to identify the CPH-material from the placebo, according to flavour or appearance.

The CPH was made by enzymatic hydrolysis of fresh frozen meat (cutting and trimmings) of Atlantic cod (*Gadus morhua),* adding the enzyme preparation Protamex^®^ (Novozymes AS, Copenhagen, Denmark) for 45 min, at 55 °C and pH 7.0. This was followed by inactivation of the enzyme, with heating to 90 °C for 15 min. The peptide containing water-soluble fraction (the hydrolysate) was separated from the indigested residue, followed by ultrafiltration and dehydration of the soluble phase to a 50% dry matter concentrate. This was spray-dried to a powder. The spray-dried CPH powder contained 89% crude protein and <0.2% fat, 0% carbohydrate, <3.0% water, 10% ash, 0.1% NaCl, 1.7% sodium and 0.07% chloride, by weight. Free amino acids accounted for 4.8% of the total amino acids in the hydrolysate, and the ratio between essential amino acids: non-essential amino acids was 0.70. Analysis of the molecular weight distribution, as well as the composition of amino acids and taurine content of the spray dried CPH powder is given in a previous publication [35].

### 2.5. Analyses of Blood Samples

Albumin, prealbumin, leucocytes, thrombocytes, hemoglobin, sodium, potassium, alanine aminotransferase, alkaline phosphatase, creatinine and aspartate aminotransferase, were analyzed at inclusion and end of study by standard accredited methods at Department of Medical Biochemistry and Pharmacology, Haukeland University Hospital (HUH), and Department of Medical Biochemistry, Ålesund Hospital.

Serum for analyses of hs-CRP, adiponectin, leptin and inflammatory markers were obtained by centrifugation of full blood at 2000× *g* at room temperature (20 °C) for 10 min after 30–60 min of coagulation, using serum separator cloth activator tubes. Hs-CRP was analyzed by standard accredited methods at the Department of Medical Biochemistry and Pharmacology, HUH. Serum adiponectin and leptin were analyzed using Human Adiponectin High Sensitivity ELISA kit (Cat. No.: RD191023100, Biovendor, Brno, Czech Republic) and Human Leptin ELISA, Clinical Range kit (Cat. No: RD191001100, Biovendor) respectively. TNF-α and IL 1β, IL 6, and IL 8 were analyzed by the Cytokine human ultrasensitive magnetic 10-plex panel for Luminex^TM^ platform (Cat.No: LHC6004M, Invitrogen, Thermo Fisher Scientific, Waltham, MA, USA). Leptin, adiponectin, as well as the inflammatory markers, were only measured and analyzed in the fasted stated.

Samples for ghrelin measurement were collected in Vacuette^®^ EDTA Aprotinin tubes (Item No: 454261, Greiner Bio-One International GmbH, Kremsmünster, Austria), added 34 μL 4-(2-aminoethyl) benzenesulfonyl fluoride hydrochloride (AEBSF) Ready-made solution (Item No: SBR00015, Sigma-Aldrich, Saint-Louis, MO, USA), right after blood sampling. Plasma for fasting and postprandial ghrelin at baseline and end of study was obtained by centrifugation of EDTA blood at 1800× *g* at −4 °C for 10 min, within 20 min after blood sampling. Ghrelin levels were analyzed using the Ghrelin Acylated Human Easy Sampling ELISA (Cat.No: RA194062500R, Biovendor) 

### 2.6. Randomization

To allocate the study participants, we used wCRF^®^, a randomization and data collection system developed by the Norwegian University of Science and Technology, Trondheim, Norway. The random assignment order was created using block randomization, and we stratified for center (Ålesund or Bergen). A person with no direct involvement in the study coded the test materials and the participants, as well as all study personnel involved in the study implementation and data handling, were blinded to group allocation.

### 2.7. Statistical Analyses

We performed statistical analyses using IBM SPSS Statistics for Windows, Version 26.0 (IBM Corp., Armonk, NY, USA) and GraphPad Prism version 8.4.2 (GraphPad Software, Inc., San Diego, CA, USA). The Shapiro-Wilk test and histograms were used to evaluate normality. For data not following a normal distribution and not improved by log-transformation, we used non-parametric tests. The Wilcoxon’s Signed Rank Test was used to investigate changes from baseline to end of study within groups, and the Independent Samples Mann Whitney U Test was used to compare changes (8 weeks—baseline) between the CPH and placebo group at end of study. These data (adiponectin, leptin, hs-CRP and inflammatory markers) are presented as median and interquartile range. A linear mixed-effects model with repeated measures was used to examine group differences over time for fasting and postprandial measurements of ghrelin. A Pearson’s correlation coefficient analysis was used to examine relationship between fasting levels of acylated ghrelin at baseline and change in body weight (kg) and BMI (kg/m^2^). The level of significance was set to *p* < 0.05. A power calculation was not done in the original study due to lack of data to base it upon [30]. Therefore, no estimation of sample size of the current measurements was done prior to the study. According to protocol, we planned to recruit 60 subjects in the study, which is a number similar to what have been reported in other supplementation studies with low doses of cod protein [15,36].

## 3. Results

### 3.1. Participant Characteristics

**Figure 1:**
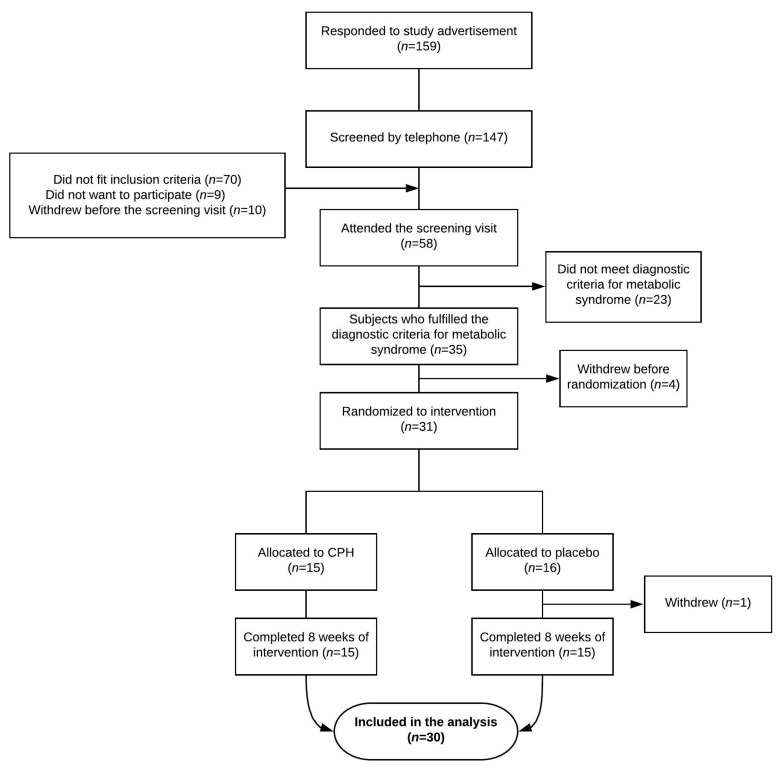
Participant flow during the study.

We screened a total of 147 participants for compliance with inclusion and exclusion criteria by telephone and invited 68 participants for a screening visit. Fifty-eight attended the screening visit and 30 participants were included and completed the intervention according to study protocol (Figure 1). Baseline characteristic are presented in Table 1.

### 3.2. Adiponectin and Leptin

**Figure 2:**
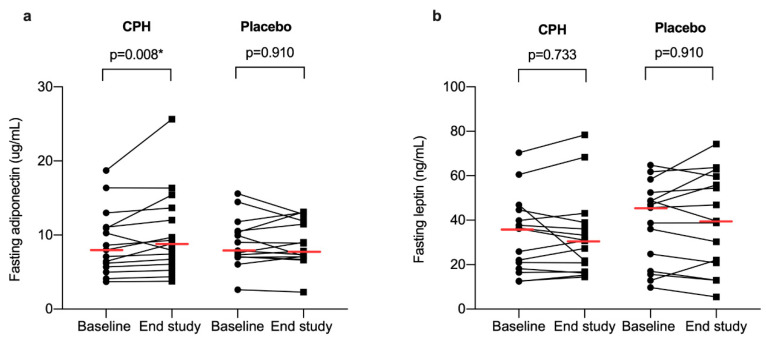
Fasting serum levels of adiponectin (**a**) and leptin (**b**) in participants with metabolic syndrome at baseline and after 8 weeks supplementation (end study) with cod protein hydrolysate (CPH) (*n* = 15) or placebo (*n* = 15). The red horizontal line shows the median levels. *p*-values within groups were calculated using the Wilcoxon’s Signed Rank Test. Statistically significant *p*-values are marked with an asterisk (*).

No statistically significant differences between the groups were observed for adiponectin (*p* = 0.806) or leptin (*p* = 0.367) at baseline. At end of study, the fasting adiponectin concentration was significantly increased within the CPH group (baseline: 7.98 (5.68, 11.06) μg/mL, end study: 8.84 (6.06, 13.7) μg/mL), *p* = 0.008), with no changes observed within the placebo group (baseline: 7.89 μg/mL (7.06, 10.53), end of study: 7.82 (7.07, 11.90) μg/mL, *p* = 0.910) (Figure 2a). The median adiponectin change (8 weeks—baseline) in the CPH group was 0.56 (0.25, 1.71) μg/mL, and −0.12 (−0.56, 1.07) μg/mL in the placebo group. When comparing the change in fasting levels of adiponectin, no statistically significant difference between groups was observed (*p* = 0.089).

No statistically significant differences in leptin levels within the CPH (baseline: 36.2 (18.2, 44.6) ng/mL, end study: 30.8 (16.8, 40.0) ng/mL, *p* = 0.733) or placebo group (baseline: 45.7 (17.1, 52.5) ng/mL end study: 39.6 (20.8, 59.6) ng/mL, *p* = 0.910) were observed from baseline to end of study (Figure 2b). The median leptin change in the CPH group was 0.30 (−3.27, 4.94) ng/mL, and 0.012 (−4.24, 8.98) ng/mL in the placebo group. When comparing the change in fasting levels of leptin, we did not observe any statistically significant difference between groups (*p* = 0.967).

### 3.3. Acylated Ghrelin Levels

**Figure 3:**
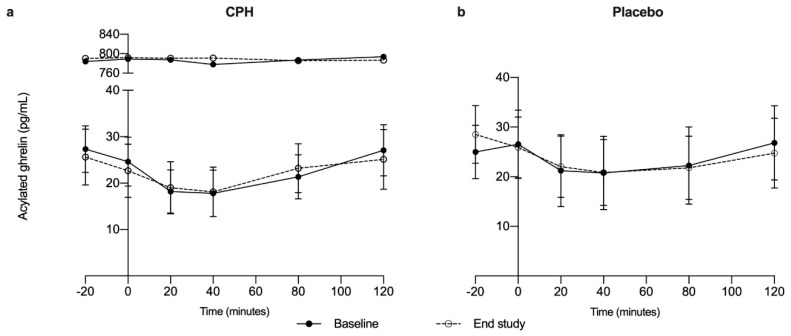

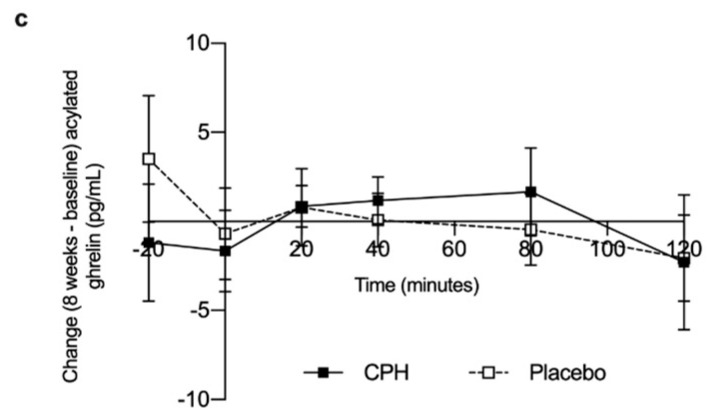
Fasting and postprandial acylated levels of ghrelin after a standardized breakfast meal at baseline (solid line) and end of study (dotted line) in participants that received supplementation with cod protein hydrolysate (CPH) (*n* = 15) (**a**) or placebo (*n* = 15) (**b**) for 8 weeks. In the CPH group, one individual had much higher levels of acylated ghrelin compared to the rest of the group and is shown as a separate segment on the graph. Graph (**c**) presents the change (calculated as 8 weeks—baseline) in acylated ghrelin during the intervention in the CPH group (solid line) compared to the placebo group (dotted line).

At baseline, the levels of acylated ghrelin were 77.8 (196.2) pg/mL in the CPH group, and 24.9 (20.8) pg/mL in the placebo group. Adjusted for time and visit, the acylated ghrelin levels were on average 51.1 pg/mL higher for the CPH group compared to placebo, but the linear mixed effects model with repeated measures analysis did not reveal any statistically significant differences between the groups (95% CI: (−54.5, 157.0), *p* = 0.330). We observed no statistically significant change in acylated ghrelin levels from baseline to end of study visit in either of the groups (overall change: 0.03 pg/mL, 95% CI: (−1.50, 1.53), *p* = 0.937) (Figure 3). There were no significant interactions between group and visit (baseline vs. end of study, *p* = 0.749), group and time (*p* = 0.693), time and visit (*p* = 0.794) or between group, visit and time (*p* = 0.853) for acylated ghrelin. 

No correlations were observed between fasting concentration of acylated ghrelin and body weight (kg) (*r* = 0.075, *p* = 0.700) or BMI (*r* = 0.172, *p* = 0.372) for the whole group at baseline (results are presented for *n* = 29, one participant excluded from the correlation analysis due to high levels). Furthermore, no correlations were observed between changes in fasting acylated ghrelin levels and changes in body weight (kg) (*r* = −0.144, *p* = 0.457) or BMI (*r* = −0.146, *p* = 0.448).

### 3.4. Inflammatory Parameters

The values of hs-CRP and inflammatory markers in serum are shown in Table 2. The serum concentrations of the cytokines were low, but detectable. 

No differences between the groups were observed for hs-CRP or any of the other inflammatory mediators at baseline. The concentrations of IL-1β, IL-6, IL-8 or TNF-α did not change during the course of the study and were not affected by supplementation with CPH (Table 2). After 8 weeks, the fasting level of hs-CRP was significantly higher within the CPH group, with no changes observed within the placebo group (Table 2). The median hs-CRP change in the CPH group was 0.1 (0.0, 2.0) mg/L, and −0.1 (−1.0, 0.55) mg/L in the placebo group. When comparing the change in fasting levels of hs-CRP, the distribution in the CPH group was significantly different from the placebo group (*p* = 0.029) (Table 2). Two participants in the placebo group were excluded from the statistical analysis of hs-CRP due to the use of lipid-lowering drugs (simvastatin, atorvastatin), because these are known to affect the levels of hs-CRP [37]. The statistical significance of results did not change if they were included in the statistical analysis of hs-CRP.

### 3.5. Adverse Effects

The blood tests taken for safety purposes were all within normal range. Seven subjects reported discomfort during the intervention period: four in the CPH group and three in the placebo group. In the CPH group, two subjects reported heartburn, two reported nausea at the beginning of the intervention period, and one reported that the supplement tasted bad and caused retching. In the placebo group, one reported itchy rash in the face, one reported nausea and one reported myalgia, but all three subjects were unsure if the symptoms were related to the intervention.

## 4. Discussion

The main objective of the present study was to investigate whether daily supplementation with low doses of CPH for 8 weeks would have an effect on circulating levels of ghrelin, adiponectin, leptin and different inflammatory markers in subjects with MetS. We hypothesized that 8 weeks supplementation with CPH would lead to a beneficial effect on circulating leptin levels and reduced inflammatory markers, as well as increased circulating levels of adiponectin and a suppressing effect on postprandial levels of ghrelin. These possibly beneficial effects were hypothesized to occur due to the presences of small peptides, mainly di- and tripeptides, suggested to be absorbed rapidly from the gastrointestinal tract and possibly influencing pathways involved in regulation of appetite and inflammation. Here, we show that 4 g of CPH for 8 weeks did not influence fasting and postprandial concentrations of acylated ghrelin or fasting levels of adiponectin, leptin or the inflammatory markers, when compared to placebo in individuals with MetS.

A daily supplementation with 4 g of CPH was sufficient to increase fasting serum adiponectin levels within the CPH group, with no change in serum levels of leptin in circulation. These results are in agreement with a previous study in healthy overweight and obese subjects receiving 2.5 g of cod protein (not hydrolyzed) for 8 weeks [23]. Observing the individual levels of adiponectin (Figure 2a), it is apparent that there are individual variations. Since the increased levels of adiponectin only were found within the CPH group and not when comparing the groups after intervention, the results should be interpreted with caution and should be explored in further studies. Still, these findings are of interest and suggests that hydrolyzed protein from lean fish may have beneficial effects on adiponectin concentration. We hypothesize that an effect may be mediated by rapidly absorbed di- and tripeptides present in the hydrolyzed supplement, containing bioactive sequences affecting metabolic pathways in the cells and thereby increasing the levels of adiponectin. Compared to the recommended daily protein intake in healthy individuals (e.g., 0.8–1.5 g protein/kg body weight/day) [38], the amount of protein in the supplement is very low, and it is plausible that it is not the increased protein content per se that is responsible for the possible metabolic effect. Bioactive peptide sequences with effect on glucose metabolism, blood pressure and lipids have been identified in other fish protein hydrolysates [39]. We have not tested for the presences of these specific known bioactive sequences in our hydrolysate, which is a limitation of the study, and the possible mechanism of action is therefore only a hypothesis.

No effects on fasting or postprandial levels of acylated ghrelin were observed after supplementation with CPH, which is in line with what we observed after giving one single dose of CPH to normal weight adults [29]. In contrast to our previous study, we used a higher dose, over a longer period, and a population with metabolic disturbances in the current study. We still did not observe any effects on circulating acylated ghrelin. In the previous study we assessed self-reported feeling of satiety and hunger [29]. An assessment of appetite was not included, which would have been an improvement of the study design seen in relation to ghrelin. By including validated questionnaires for reporting appetite and a free eating lunch at each study visit, we would be able to calculate energy intake and assess whether supplementation with CPH for 8 weeks led to lower energy intake, and thereby suggesting a suppressing effect on appetite.

Fish has been proposed to have anti-inflammatory properties, and reduced levels of CRP have been linked to fish consumption [13]. A diet with cod protein reduced the levels of hs-CRP in insulin-resistant subjects [14], whereas others have reported of no effect on CRP levels or other inflammatory markers after a high intake of cod in normal-weight [40] or overweight subjects [41]. No effect on CRP levels was observed in an intervention study with overweight and obese subjects supplemented with similar amount of cod protein (intact protein) as used in the current trial [15]. We observed higher levels of hs-CRP after supplementation with CPH for 8 weeks, when compared to placebo, but did not observe any changes in other inflammatory markers. An increased level of CRP was reported in elderly subjects living in a nursing home setting given 5.2 g of fish protein (blue whiting) for 6 weeks (compared to placebo) [42], however, this group is not comparable to our study population. Overall, the higher levels of hs-CRP in the CPH group is difficult to interpret. It is possible that the small sample size might have influenced the results, or that some individuals may have had some on-going low-grade inflammation without disclosing a problem. When comparing the overall fasting levels of hs-CRP in both groups in our study with a previous study by Delongui et al. [43], our levels are similar to the levels reported in obese subjects and higher compared to normal weight subjects, emphasizing how BMI may affect CRP levels [43].

There are certain strengths and limitations to the study. Firstly, the randomized, double-blinded design is a strength. A cross-over design would potentially have been an even better design, with each subject serving as his/her own control and allowing us to recruit fewer subjects without compromising the strength of the study [44]. Since this would have resulted in a long intervention period and possibly higher dropout rate, we chose to conduct the current study as a parallel group study. Secondly, adjusting the CPH dosage to the body weight of each participant could have further strengthened the design, as it would reduce the effect of variation in body weight. It was not practically feasible, and we chose to use a dose similar to what has previously been effective [15,36,45]. Thirdly, the lack of a power analysis is a weakness when interpreting the data, but there was no relevant data to base such calculation upon. As we had difficulties recruiting, we did not reach our target of 60 subjects (with 30 individuals in each treatment arm), which might have further affected the results. In particular we had difficulties recruiting males and a predominance of female participants were included in the study. Due to limited resources and time constraint, the inclusion of new participants was stopped in September 2019. It cannot be ruled out that our results might have changed in a larger study, so replication from future studies are necessary to examine the robustness of our findings, in particular related to fasting levels of serum adiponectin and hs-CRP.

## 5. Conclusions

To conclude, our study showed that a daily supplement of 4 g of CPH for 8 weeks was not sufficient to affect fasting or postprandial levels of ghrelin, or fasting levels of adiponectin, leptin or inflammatory markers in overweight and obese subjects with MetS.

## Figures and Tables

**Table 1:** Characteristics of the participants in the cod protein hydrolysate (CPH) group (*n* = 15) and the placebo group (*n* = 15) before intervention.

Variable	CPH		Placebo		*p*-Value
	Mean	SD	Mean	SD	
Gender, female/male	11/4		13/2		0.651
Age, years	52.8	6.26	53.4	6.83	0.804
Body weight, kg	96.5	12.8	93.4	12.2	0.509
BMI, kg/m^2^	32.7	2.24	32.4	3.25	0.751
Waist circumference	107.6	9.72	105.7	10.7	0.630
Systolic BP, mmHg	136.9	15.9	138.5	15.1	0.756
Diastolic BP, mmHg	88.2	10.1	86.7	6.44	0.702
Energy intake, kcal	1882	485	1812	386	0.668
Protein intake, g/kg BW/day	0.9	0.2	0.9	0.3	0.992
Antihypertensive, *n*	5		9		−
Smokers, *n*	1		2		−

SD, standard deviation; BMI, body mass index; BP, blood pressure, BW; body weight. Results are presented as mean ± SD. Groups were compared at baseline using Independent Samples *t*-test.

**Table 2:** The concentration of inflammatory markers in serum samples collected before and after 8 weeks supplementation with cod protein hydrolysate (CPH) (*n* = 15) or placebo (*n* = 15).

		Baseline		8 Weeks		*p*-Value ^1^	*p*-Value ^2^
		Median	25th, 75th Percentile	Median	25th, 75th Percentile		
**Hs-CRP, mg/L**							0.029 *
	CPH	4.0	1.0, 4.0	4.0	2.0, 6.0	0.021 *	
	Placebo	3.0	1.5, 7.0	3.0	2.0, 7.0	0.389	
**IL-1** **β** **, pg/mL**							0.567
	CPH	0.13	0.13, 0.41	0.13	0.13, 0.41	0.574	
	Placebo	0.13	0.13, 0.41	0.13	0.13, 0.41	0.589	
**IL-6, pg/mL**							0.935
	CPH	1.04	0.52, 1.77	0.90	0.74, 1.48	0.394	
	Placebo	1.19	0.75, 1.34	1.04	0.59, 1.34	0.396	
**IL-8, pg/mL**							0.174
	CPH	15.8	11.9, 20.3	17.6	14.0, 22.5	0.096	
	Placebo	18.1	15.1, 26.9	16.7	12.2, 23.0	0.281	
**TNF-α, pg/mL**							0.935
	CPH	0.57	0.22, 0.93	0.93	0.22, 0.93	0.573	
	Placebo	0.22	0.11, 0.93	0.57	0.22, 0.93	0.280	

Hs-CRP; high-sensitivity C-reactive protein; IL, interleukin; TNF-α, tumor necrosis factor alpha. The data are presented ad median and interquartile range. (1) *p*-values within groups are based on Wilcoxon’s Signed Rank Test. (2) *p*-values between groups are based on Mann Whitney U Test. Statistically significant *p*-values are marked with an asterisk (*).
